# Smoking-Attributable Health Care Expenditures for US Adults With Chronic Lower Respiratory Disease

**DOI:** 10.1001/jamanetworkopen.2024.13869

**Published:** 2024-05-30

**Authors:** Dian Gu, Hai-Yen Sung, Carolyn S. Calfee, Yingning Wang, Tingting Yao, Wendy Max

**Affiliations:** 1 Institute for Health & Aging, School of Nursing, University of California, San Francisco; 2 The Center for Tobacco Control Research and Education, University of California, San Francisco; 3 Division of Pulmonary and Critical Care Medicine, Department of Medicine, University of California, San Francisco

## Abstract

**Importance:**

Cigarette smoking is a primary risk factor for chronic lower respiratory disease (CLRD) and is associated with worse symptoms among people with CLRD. It is important to evaluate the economic outcomes of smoking in this population.

**Objective:**

To estimate smoking prevalence and cigarette smoking-attributable health care expenditures (SAHEs) for adults with CLRD in the US.

**Design, Setting, and Participants:**

This cross-sectional study used data from the 2014-2018 and 2020 National Health Interview Surveys (NHIS) and the 2020 Medical Expenditure Panel Survey. The final study population, stratified by age 35 to 64 years and 65 years or older, was extracted from the 2014-2018 NHIS data. The data analysis was performed between February 1 and March 31, 2024.

**Exposures:**

Cigarette smoking, as classified into 4 categories: current smokers, former smokers who quit less than 15 years ago, former smokers who quit 15 or more years ago, and never smokers.

**Main Outcomes and Measures:**

Smoking-attributable health care expenditures were assessed using a prevalence-based annual cost approach. Econometric models for the association between cigarette smoking and health care utilization were estimated for 4 types of health care services: inpatient care, emergency department visits, physician visits, and home health visits.

**Results:**

In the 2014-2018 NHIS study sample of 13 017 adults, 7400 (weighted 62.4%) were aged 35 to 64 years, 5617 (weighted 37.6%) were 65 years or older, and 8239 (weighted 61.9%) were female. In 2020, among 11 211 222 adults aged 35 to 64 with CLRD, 3 508 504 (31.3%) were current smokers and 3 496 790 (31.2%) were former smokers. Total SAHEs in 2020 for this age group were $13.6 billion, averaging $2752 per current smoker and $1083 per former smoker. In 2020, 7 561 909 adults aged 65 years or older had CLRD, with 1 451 033 (19.2%) being current smokers and 4 104 904 (54.3%) being former smokers. Total SAHEs in 2020 for the older age group were $5.3 billion, averaging $1704 per current smoker and $682 per former smoker. In sum, SAHEs for adults with CLRD aged 35 years or older amounted to $18.9 billion in 2020.

**Conclusions and Relevance:**

In this cross-sectional study of adults with CLRD, cigarette smoking was associated with a substantial health care burden. The higher per-person SAHEs for current smokers compared with former smokers suggest potential cost savings of developing targeted smoking cessation interventions for this population.

## Key Points

**Question:**

What is the association of smoking with health care expenditures among US adults with chronic lower respiratory disease?

**Findings:**

In this cross-sectional study of 13 017 adults with chronic lower respiratory disease in 2020, a weighted 31.3% and 31.2% of those aged 35 to 64 years were current and former smokers, respectively, with annual smoking-attributable health care expenditures (SAHEs) of $13.6 billion, or $2752 per current smoker and $1083 per former smoker. Among those 65 years or older, a weighted 19.2% were current smokers and 54.3% were former smokers, with annual SAHEs of $5.3 billion, or $1704 per current smoker and $682 per former smoker.

**Meaning:**

The high SAHEs found in this study suggest the importance of targeted cessation interventions for this population.

## Teaser

This cross-sectional study estimates cigarette smoking prevalence and smoking-attributable health care expenditures for US adults with chronic lower respiratory disease.

## Introduction

Chronic lower respiratory disease (CLRD) is a group of progressive, debilitating respiratory conditions that generally includes chronic obstructive pulmonary disease (COPD)—including chronic bronchitis and emphysema—and asthma. Millions of people are affected by CLRD, which is a major cause of mortality and disability worldwide.^1^ According to US National Health Interview Surveys (NHIS), 4.6% of adults reported having COPD, and 3.7% reported having asthma episodes or attacks in 2022.^2,3^ Chronic lower respiratory disease caused 142 342 deaths in 2021, ranking as the sixth leading cause of death in the US^4^ and accounting for approximately 3 million years lived with disability in 2019.^5^ Chronic lower respiratory disease is costly, imposing a substantial economic burden on individuals and society in the US.^6,7,8,9^ A recent study estimated that US health care spending totaled $34.3 billion for COPD and $35.5 billion for asthma in 2016.^10^

Cigarette smoking is the dominant risk factor for deaths and disability from COPD^1,11,12^ and is associated with worse symptoms among people with COPD.^13^ Evidence suggests an association between active smoking and exacerbation of asthma in adults.^14^ However, little is known about smoking prevalence in people with CLRD. Several studies estimated smoking rates for people with COPD and people with asthma separately, but these studies were conducted using data from a decade ago.^15,16,17^ More recent estimates of smoking prevalence across the entire CLRD spectrum are needed to determine smoking’s impact on this vulnerable group.

Given the high health care expenditures and negative health outcomes associated with smoking for people with CLRD, it is important to evaluate the health-related economic costs attributable to cigarette smoking among people with CLRD. Previous studies that assessed the health care costs of smoking in the US have focused on the general population at the national or state level^18,19,20,21^ or subpopulations including Medicaid and Medicare enrollees, military veterans, and racial minority groups,^22,23,24,25,26^ but little work has been done for people with CLRD. One exception is a recent study that estimated that in 2016, $15.3 billion of the total $34.3 billion spent on treating COPD was attributed to cigarette smoking.^10^ However, that study did not estimate smoking-attributable health care expenditures (SAHEs) of treating all diseases for people with COPD and/or asthma.

The aim of this study is to fill the literature gap by providing more current national estimates of cigarette smoking prevalence and SAHEs among adults with CLRD in the US. These estimates may be useful for developing targeted tobacco control measures to reduce the burden of CLRD and for providing up-to-date information to support the development of appropriate interventions for this subpopulation.

## Methods

This cross-sectional study was certified as exempt from review and informed consent by the Human Research Protection Program Institutional Review Board at the University of California, San Francisco. This study followed the Strengthening the Reporting of Observational Studies in Epidemiology (STROBE) reporting guideline.

We used a prevalence-based annual cost approach to estimate an econometric model of the association between cigarette smoking and health care utilization. From the estimated model and smoking prevalence, the smoking-attributable fraction (SAF)—the proportion of excess utilization attributed to smoking—was calculated using an excess utilization approach.^20,22,26,27,28^ We multiplied this SAF by annual total health care expenditures for CLRD to obtain SAHEs for CLRD.

### Data Sources

We analyzed cross-sectional data from the 2014-2018 and 2020 NHIS and the 2020 Medical Expenditure Panel Survey (MEPS). The NHIS is a nationally representative household survey of the civilian noninstitutionalized US population, collecting information on respondents’ sociodemographic characteristics and a wide range of health measures, health care access and utilization, and risk behaviors.^29^ The MEPS is a nationally representative survey of the civilian noninstitutionalized US population, containing detailed information on individuals’ health conditions, health insurance coverage, health care utilization, and expenditures.^30^ The 2020 MEPS sample included 3 overlapping panels drawn from the 2017, 2018, and 2019 NHIS samples, respectively.^30^

### Study Samples

Because COPD is uncommon among younger people,^10,31^ we focused on adults aged 35 years or older. Similar to previous studies,^2,3,16^ we identified adults with CLRD from the NHIS data based on affirmative answers to any of the following questions: “Have you ever been told by a doctor or other health professional that you had chronic obstructive pulmonary disease (COPD), emphysema, or chronic bronchitis” (3 separate questions prior to 2019), and “During the past 12 months, have you had an episode of asthma or an asthma attack?” Adults with CLRD were identified in the 2020 MEPS data if they affirmatively answered the same questions about emphysema, chronic bronchitis, and asthma or had any medical event (hospital stay, outpatient visit, emergency department [ED] visit, home health episode, prescribed medication purchase, or medical provider visit) in the survey year due to a CLRD diagnosis (*International Statistical Classification of Diseases and Related Health Problems, Tenth Revision* codes J41-J47).^10^

### Outcome Measures

Cigarette smoking status was classified as current smokers, former smokers who quit more than 15 years ago, former smokers who quit 15 or more years ago, and never smokers. Current smokers are those who smoked 100 or more cigarettes during their lifetime and now smoke cigarettes every day or some days.^32^ Former smokers are those who smoked 100 or more cigarettes during their lifetime but do not smoke now.^32^ Never smokers are those who never smoked any cigarette or who smoked less than 100 cigarettes during their lifetime.^32^

Health care utilization and expenditures were estimated for inpatient care, ED visits, physician visits, and home health visits. Inpatient care was measured by hospital nights during the past 12 months. Health status was constructed as an ordinal variable based on the question, “Would you say your health, in general, is excellent, very good, good, fair, or poor,” with 1 indicating excellent and 5 indicating poor.

### Covariates in Econometric Model

Sociodemographic characteristics included sex (female and male), age (years), self-reported race and ethnicity (Hispanic, non-Hispanic Asian, non-Hispanic Black, non-Hispanic White, and non-Hispanic other [including non-Hispanic American Indian or Alaska Native, race group not releasable, and multiple races]), educational level (less than high school, high school graduate, some college, and college degree or greater), poverty status (poor [<100% of the federal poverty level (FPL)], low income [100%-199% FPL], middle income [200%-399% FPL], high income [≥400% FPL], and unknown), marital status (married; widowed, separated, or divorced; living with partner; and never married), region of residence (Northeast, Midwest, South, and West), and health insurance coverage (private coverage, Medicaid or other public insurance, Medicare only, other public coverage, and uninsured). Race and ethnicity were self-reported through 2 separate survey questions in the NHIS. Individuals who self-reported as Hispanic or Latino were classified as Hispanic regardless of their race selection.^33^ Those who did not identify themselves as Hispanic were classified based on their self-reported race.^33^ Because of racial and ethnic disparities in health care spending,^34^ we included race and ethnicity as a covariate in the study. Other risks included body weight (underweight [body mass index (BMI) as measured by weight in kilograms divided by height in meters squared, <18.5], normal weight [BMI, 18.5-24.9], overweight [BMI, 25.0-29.9], obesity [BMI, ≥30.0], and unknown), and binge drinking (yes, no, and unknown).

### Smoking-Attributable Health Care Utilization Analysis

To estimate the association of cigarette smoking with health care utilization among adults with CLRD, we used a 9-equation econometric model adapted from the models that Max and colleagues have developed and refined over the past 3 decades.^20,22,26,27,28^ Details of the specification and estimation method for the 9 equations are reported in the eMethods in Supplement 1.

The model was estimated separately by age group (35-64 years and ≥65 years). On the basis of the estimation model, we calculated the predicted utilization for smokers and for hypothetical never-smoking smokers, who were identical to smokers in every way except that they were assumed to have never smoked cigarettes. The difference between these 2 sets of predictions was the excess utilization of smoking. The ratio of the mean predicted utilization for smokers (or hypothetical never-smoking smokers) to the mean predicted utilization for never smokers is the relative risk (RR) of health care utilization for smokers (or hypothetical never-smoking smokers). Assuming that the RRs would remain constant over time from 2014 to 2020, we calculated the 2020 SAF of health care utilization by applying these RRs along with the smoking prevalence estimated from the 2020 NHIS to the SAF equation^21,27^ (equation 10 in the eMethods in Supplement 1). In the main analysis, we set the negative SAFs to 0.

### Estimation of SAHEs

We calculated SAHEs for each age group and type of health service by multiplying the appropriate SAF by the corresponding 2020 health care expenditures for adults with CLRD estimated from the 2020 MEPS data. The sum of the expenditures for the 2 age groups is the total SAHEs for adults aged 35 years or older in 2020. Expenditures include all payment sources.

### Final Sample Size

The 2020 smoking prevalence among US adults with CLRD was estimated using the 2020 NHIS data, which contained 2430 respondents with CLRD older than 35 years. After excluding 64 respondents with missing data on smoking status (2.6%), the final study sample was 2366 adults.

To estimate the model for the association between cigarette smoking and health care utilization, we used pooled data from the 2014-2018 NHIS, which contained 13 439 respondents with CLRD aged 35 years or older. Because the NHIS has stopped collecting health care utilization information except ED visits since 2019, the 2019-2020 NHIS data were not included. After excluding respondents with incomplete information for smoking status, health care utilization, health status, and covariates (422 [3.1%]), the final study sample included 13 017 adults. Based on statistical guidance,^35,36^ the small missing sample rate would be inconsequential to the model estimation.

We used the 2020 MEPS to calculate total annual expenditures by type of health care services for adults with CLRD. The data contained 1786 respondents aged 35 years or older with CLRD. We also conducted a sensitivity analysis using the SAF values derived from the econometric model, even if the value was negative, to calculate SAHEs.

### Statistical Analysis

Statistical analyses were performed between February 1 and March 31, 2024. The analyses accounted for the sampling weight and complex survey design of the NHIS and MEPS. The econometric model for the association between smoking and health care utilization was estimated using NLOGIT, version 3.0 (Econometric Software, Inc), and all other analyses were performed using SAS, version 9.4 (SAS Institute Inc). Statistical tests to determine whether the estimated coefficients from the econometric model were significant are reported in the eMethods in Supplement 1. Statistical significance was set at a 2-tailed *P* < .05.

## Results

### Characteristics of the 2014-2018 NHIS Study Sample

Among the study sample of 13 017 adults with CLRD from the 2014-2018 NHIS data, a weighted 62.4% were aged 35 to 64 years and 37.6% were aged 65 years or older; 61.9% were female and 38.1% were male; and 8.8% were Hispanic, 2.4% non-Hispanic Asian, 11.0% non-Hispanic Black, 74.9% non-Hispanic White, and 2.9% non-Hispanic other (Table 1). Health care utilization of the study sample is shown in Table 2.

**Table 1.** Sample Characteristics From the National Health Interview Surveys, 2014-2018

Characteristic	No. of adults with CLRD (weighted %) (N = 13 017)
Age group, y	
35-64	7400 (62.4)
≥65	5617 (37.6)
Sex	
Female	8239 (61.9)
Male	4778 (38.1)
Race and ethnicity	
Hispanic	1056 (8.8)
Non-Hispanic Asian	278 (2.4)
Non-Hispanic Black	1566 (11.0)
Non-Hispanic White	9652 (74.9)
Non-Hispanic other^a^	465 (2.9)
Educational level	
Less than high school	2456 (17.6)
High school graduate	3714 (28.6)
Some college	4142 (31.2)
College degree or higher	2705 (22.6)
Poverty status, federal poverty level	
<100%	2655 (16.1)
100%-199%	3052 (21.8)
200%-399%	3398 (26.6)
≥400%	3181 (29.6)
Unknown	731 (5.9)
Marital status	
Married	4969 (51.2)
Widowed, divorced, or separated	5984 (33.9)
Living with partner	543 (5.4)
Never married	1521 (9.5)
Region of residence	
Northeast	2172 (17.1)
Midwest	3042 (24.4)
South	4895 (38.8)
West	2908 (19.7)
Health insurance coverage	
Private coverage^b^	6269 (51.9)
Medicaid or other public insurance^c^	2726 (18.6)
Medicare only	1793 (12.7)
Other coverage^d^	1485 (10.8)
Uninsured	744 (6.0)
Body weight status	
Underweight	344 (2.4)
Normal	3156 (23.2)
Overweight	3812 (29.4)
Obesity	5323 (41.8)
Unknown	382 (3.2)
Binge drinking status	
Yes	1999 (16.3)
No	10 823 (82.1)
Unknown	195 (1.6)
Health status	
Excellent	907 (7.9)
Very good	2477 (19.7)
Good	4160 (32.2)
Fair	3585 (26.2)
Poor	1888 (14.0)

Abbreviation: CLRD, chronic lower respiratory disease.

^a^ Includes non-Hispanic American Indian or Alaska Native, race group not releasable, and multiple races.

^b^ Defined as any private insurance plan for individuals younger than 65 years and for individuals 65 years or older with both Medicare and any private insurance plan.

^c^ For individuals younger than 65 years, this category includes those not classified in private coverage but who have Medicaid or other state-sponsored health plans regardless of Medicare coverage; thus, this category includes dual Medicare and Medicaid coverage. For individuals 65 years or older, this category includes those not classified in private coverage but who have both Medicaid and public coverage; thus, this category includes dual Medicare and Medicaid coverage.

^d^ For individuals younger than 65 years, this category includes those not classified in the above 2 categories but who have any type of military or other government program coverage. For individuals 65 years or older, this category includes those not classified in the first 3 categories of this variable but who have both Medicare and coverage of military medical insurance or Indian Health Service and those who have public coverage only, military coverage only, or Indian Health Service coverage only.

**Table 2.** Cigarette Smoking Status and Health Care Utilization of the Study Sample, National Health Interview Surveys, 2014-2018

Variable	No. of adults with CLRD (weighted %)	
	Aged 34-64 y (n = 7400)	Aged ≥65 y (n = 5617)
**Cigarette smoking status**		
Current smoker	2580 (32.4)	1029 (17.4)
Former smoker	1924 (26.0)	2823 (50.9)
<15 y	1203 (16.0)	1045 (18.4)
≥15 y	721 (10.0)	1778 (32.5)
Never smoker	2896 (41.5)	1765 (31.7)
**Health care utilization**		
Hospital nights in the past 12 mo		
Yes, having ≥1	1395 (17.1)	1484 (25.6)
Mean No. of nights among those with ≥1 night (SEM)	9.2 (0.6)	8.8 (0.4)
ED visits in the past 12 mo		
Yes, having ≥1 visit	2991 (38.1)	2140 (37.9)
Mean No. of visits among those with ≥1 visit (SEM)	2.8 (0.1)	2.2 (0.1)
Physician visits in the past 2 wk		
Yes, having ≥1 visit	2816 (36.3)	2429 (42.8)
Mean No. of visits among those with ≥1 visit (SEM)	1.7 (0.0)	1.6 (0.0)
Home health visits in the past 2 wk		
Yes, having ≥1 visit	259 (2.6)	466 (7.5)
Mean No. of visits among those with ≥1 visit (SEM)	5.3 (0.3)	5.6 (0.2)

Abbreviations: CLRD, chronic lower respiratory disease; ED, emergency department; SEM, standard error of the mean.

### Adult Population With CLRD and Their Smoking Prevalence in 2020

The 2020 MEPS indicated that 18 773 131 US adults aged 35 years or older had CLRD, including 11 211 222 (59.7%) aged 35 to 64 and 7 561 909 (40.3%) aged 65 years or older, and the prevalence of CLRD was higher among those aged 65 years or older compared with those aged 35 to 64 (13.0% vs 9.1%, respectively) (eTable 1 in Supplement 1). Total health care expenditures for these adults were $202.2 billion.

Among 1174 adults aged 35 to 64 with CLRD from 2020 NHIS data, a weighted 31.3% were current smokers and 31.2% were former smokers (Table 3). In contrast, among 1192 adults aged 65 years or older with CLRD, a weighted 19.2% were current smokers and 54.3% were former smokers.

**Table 3.** Cigarette Smoking Status of the Study Sample, National Health Interview Survey, 2020

Cigarette smoking status	No. of adults with CLRD (weighted %)	
	Aged 35-64 y (n = 1174)	Aged ≥65 y (n = 1192)
Current smoker	359 (31.3)	207 (19.2)
Former smoker	361 (31.2)	654 (54.3)
<15 y	226 (20.1)	248 (22.3)
≥15 y	135 (11.1)	406 (32.0)
Never smoker	454 (37.5)	331 (26.5)

Abbreviation: CLRD, chronic lower respiratory disease.

### SAF of Health Care Utilization

Estimated results from the econometric model are shown in eTable 2 in Supplement 1. The SAFs were calculated based on the estimated econometric models and smoking prevalence rates (Table 4). For those aged 35 to 64, total SAFs for all current and former smokers were 22.4% for home health visits, 19.0% for inpatient care, 9.2% for ED visits, and 7.1% for physician visits. The SAF for current smoking was more than twice that for former smoking for each type of health care utilization. On the other hand, those aged 65 years or older had smaller SAFs for each category of smokers and each type of health care utilization compared with those aged 35 to 64 except for the former smokers who quit less than 15 years ago, and the SAF for current smoking was slightly smaller than that for former smoking for each type of health care utilization. The SAFs for former smokers who quit 15 or more years ago in the older group were originally estimated as negative values, suggesting that this group had lower health care utilization compared with never smokers. While many studies have found that smoking cessation can greatly reduce the risk of tobacco-associated morbidity and mortality at all ages,^14,37,38,39,40^ it is not clear why their risk would be reduced to a lower level than that of never smokers. Following a previous study,^41^ we set negative SAF values to 0 in the main analysis.

**Table 4.** Smoking-Attributable Fraction (SAF) of Health Care Utilization by Type of Health Services, Smoking Status, and Age Group, 2020

Cigarette smoking status	SAF, %^a^			
	Inpatient care	ED visits	Physician visits	Home health visits
**Aged 35-64 y**				
Current smokers	13.7	6.6	4.9	15.5
Former smokers	5.3	2.6	2.2	5.9
<15 y	4.6	2.3	1.9	5.1
≥15 y	0.7	0.3	0.3	0.8
All smokers	19.0	9.2	7.1	22.4
**Age ≥65 y**				
Current smokers	3.9	2.7	1.6	3.3
Former smokers	4.2	3.0	1.8	4.2
<15 y	4.2	3.0	1.8	4.2
≥15 y	0.0^b^	0.0	0.0	0.0^b^
All smokers	8.1	5.7	3.4	7.5

Abbreviation: ED, emergency department.

^a^ Derived based on the estimated econometric models shown in eTable 2 in Supplement 1 and the 2020 cigarette smoking prevalence rates shown in Table 3.

^b^ The original SAF values that were calculated based on the estimated econometric model were −1.9%, −1.3%, −0.8%, and −2.4% for inpatient care, ED visits, physician visits, and home health visits, respectively. Because a negative SAF suggests that former smokers possess a reduced morbidity risk compared with never smokers, which is against the evidence linking smoking and health, we assume that these SAFs are 0.

### SAHEs

Annual SAHEs among adults aged 35 years or older with CLRD were $18.9 billion in 2020, including $13.6 billion (72.0% of the total) for those aged 35 to 64 and $5.3 billion (28.0% of the total) for those aged 65 years or older (Table 5). Among those aged 35 to 64 years, 53.5% of the SAHEs ($7.3 billion) were for inpatient care. Of the total SAHEs, 71.3% ($9.7 billion) were for current smokers and 28.7% ($3.9 billion) were for former smokers. The per-smoker SAHEs were derived by dividing the total SAHEs by the number of smokers with CLRD for each smoking status category (eTable 3 in Supplement 1). The per-smoker SAHEs averaged $2752 per current smoker and $1083 per former smoker. Stratified by time since quitting, per-smoker SAHEs were $1502 for former smokers who quit less than 15 years ago and $412 for those who quit 15 or more years ago.

**Table 5.** Annual Cigarette SAHEs in 2020 by Type of Health Services, Smoking Status, and Age Group for US Adults With CLRD

Cigarette smoking status	SAHE by type of health services (millions), $^a^^,^^b^				Total SAHE in billions, $^a^	Per-smoker SAHE, $^a^^,^^c^
	Inpatient care	ED visits	Physician visits	Home health visits		
**Aged 35-64 y**						
Current smokers	5240	419	2936	1062	9.7	2752
Former smokers	2017	166	1318	400	3.9	1083
<15 y	1765	146	1131	346	3.4	1502
≥15 y	252	20	187	54	0.5	412
All smokers (%)	7257 (53.5)	585 (4.3)	4254 (31.4)	1462 (10.8)	13.6	2010
**Aged ≥65 y**						
Current smokers	1211	63	636	563	2.5	1704
Former smokers	1290	69	715	726	2.8	682
<15 y	1290	69	715	726	2.8	1662
≥15 y	0	0	0	0	0	0
All smokers (%)	2501 (47.4)	132 (2.5)	1351 (25.6)	1289 (24.4)	5.3	949

Abbreviations: CLRD, chronic lower respiratory disease; ED, emergency department; SAHE, smoking-attributable health care expenditure.

^a^ In 2020 dollars.

^b^ Derived by multiplying the smoking-attributable fraction values (shown in Table 4) by annual health care expenditures among adults with CLRD (shown in eTable 1 in Supplement 1) for each type of health service and age group.

^c^ Derived by dividing total SAHE by the number of smokers with CLRD for each smoking status category (shown in eTable 3 in Supplement 1).

Among respondents aged 65 years or older, 47.4% of the SAHEs ($2.5 billion) were for inpatient care. Of the total SAHEs, 52.8% ($2.8 billion) were for former smokers, and 47.2% ($2.5 billion) were for current smokers. The per-smoker SAHEs averaged $1704 for current smokers, $1662 for former smokers who quit less than 15 years ago, and $682 for former smokers regardless of time since quitting. The sensitivity analysis results showed that using the negative SAFs for former smokers who quit 15 or more years ago in the older group would result in a total SAHE of $17.5 billion (eTable 4 in Supplement 1).

## Discussion

To our knowledge, this is the first study to estimate health care costs of cigarette smoking for people with CLRD in the US. We found that 18.8 million adults aged 35 years or older had CLRD in 2020 and that their total health expenditures were $202.2 billion, of which $18.9 billion were attributed to cigarette smoking. We also found that among these adults with CLRD, 31.3% of those aged 35 to 64 and 19.2% of those aged 65 years or older were current smokers in 2020, with smoking rates more than twice as high as those for the general population of US adults.^42^ Our results suggest that many people with CLRD continue to smoke, and smoking among this vulnerable population was associated with high costs to the health care system. These findings shed light on the economic consequence of smoking for people who have CLRD and highlight the importance of developing intervention strategies to increase smoking cessation for this vulnerable population.

We found that per-person SAHEs were greater for current than for former smokers in both age groups, suggesting that increasing cessation among smokers with CLRD may reduce health care costs. It is noteworthy that per-person SAHEs for former smokers who quit 15 or more years ago were substantially lower than per-person SAHEs for those who quit less than 15 years ago in both age groups. Our finding of 0 per-person SAHEs among former smokers of 15 or more years suggests that their health care utilization and expenditures decreased to the level of never smokers’ after 15 years of quitting. Many studies have shown that smoking cessation can greatly reduce the risk of tobacco-associated morbidity and mortality at all ages, even for older smokers.^14,37,38,39,40^ Specifically, evidence has shown that smoking cessation is associated with a reduction in COPD hospitalization risk^43^ and with a variety of health benefits, such as improved lung function.^44^ Our findings offer additional evidence of the association between years since quitting and SAHEs. These estimates may be useful for economic evaluations, such as cost-effectiveness analyses of smoking cessation interventions among smokers with CLRD. Future research is needed to more comprehensively analyze the time course of health care expenditure reduction following cessation for smokers with CLRD.

Among adults with CLRD, the estimated SAFs ranged from 7.1% to 22.4% for those aged 35 to 64 and 3.4% to 8.1% for those 65 years or older depending on health service type. A review of US cost-of-smoking studies that were published prior to 1994 concluded that the SAFs of health care costs for all service types ranged between 6.0% and 11.8%.^45^ A more recent article estimated that the SAFs of health care spending were 11.7% for all service types, 16.4% for inpatient care, 6.0% for noninpatient care (including outpatient services, physician and clinical services, and other professional services), and 13.4% for prescription medications during 2010-2014.^19^ However, the SAFs reported in both these articles are for the general population of US adults aged 18 years or older. There is no research estimating the SAFs specifically for adults with CLRD. Our estimates establish a baseline for comparison in future research estimating the SAFs for people with CLRD or other smoking-related diseases.

### Limitations

This study has some limitations. First, smoking status was based on self-report and lacked biochemical verification, potentially resulting in classification errors. However, self-reported measurements of smoking status generally have high sensitivity and specificity compared with the results of biochemical validation.^46^ Hence, the misclassification rate should be low. Second, self-reported health care utilization may have recall bias. Third, we posited an equivalence between the RR of health care expenditures and the RR of health care utilization. Our estimates could be biased if this postulate is invalid. Fourth, we were not able to account for health care services, such as nursing home care, prescription drugs, durable medical equipment, and nondurable medical products (eg, over-the-counter medications), due to data limitations.^10^ This limitation may cause an underestimation of the SAHEs, which is particularly important for adults aged 65 years or older with CLRD who may have multiple comorbidities and more health care needs beyond what is captured by the 4 types of services we examined. Fifth, we were unable to estimate expenditures attributable to exposure to secondhand smoke, which also may cause underestimation of SAHEs. Sixth, because of data limitations, we could not control for risk factors, such as cannabis and e-cigarette use, that may moderate the association between cigarette smoking and health care expenditures for people with CLRD. Further investigation is needed to incorporate a broader range of risk factors with valid measurements. Finally, this study did not consider indirect costs of smoking, such as productivity loss due to smoking-attributable premature death and employee absenteeism or presenteeism.^12,28,47,48^ Future research is needed to estimate the full scope of the economic burden of smoking for this population.

## Conclusions

In this cross-sectional study of adults with CLRD, health care expenditures attributable to cigarette smoking were substantial. Because many people with CLRD continued to smoke, our findings suggest potential cost savings of developing targeted smoking cessation interventions for this population.

## Notes

Supplement 1. **eMethods.** Supplemental Methods
eReferences
**eTable 1.** Number and Percentage of US Adults (Aged 35+ Years) Identified to Have Chronic Lower Respiratory Disease (CLRD) in 2020 and Their Health Care Expenditures in 2020 by Type of Services and Age Group **eTable 2.** Estimated Coefficients of the Econometric Models for the Impact of Cigarette Smoking on Health Care Utilization Among US Adults Aged 35+ Years With Chronic Lower Respiratory Disease: National Health Interview Survey, 2014-2018 **eTable 3.** Number of Smokers Among US Adults (Aged 35+ Years) With Chronic Lower Respiratory Disease (CLRD) in 2020 by Age Group **eTable 4.** Sensitivity Analysis Which Allows for the Negative SAF Values: Annual Cigarette Smoking-Attributable Health Care Expenditures (SAHEs) in 2020 by Type of Health Services, Smoking Status, and Age Group for US Adults Aged 35+ Years With Chronic Lower Respiratory Disease (CLRD)

Supplement 2.
Data Sharing Statement
