# Supplementary material for: Smoking-Attributable Health Care Expenditures for US Adults With Chronic Lower Respiratory Disease
Source: JAMA Netw Open. 2024 May 30;7(5):e2413869. doi: 10.1001/jamanetworkopen.2024.13869 (PMC11140527; doi:10.1001/jamanetworkopen.2024.13869)
Supplement: Supplement 1. — eMethods. Supplemental Methods eReferences eTable 1. Number and Percentage of US Adults (Aged 35+ Years) Identified to Have Chronic Lower Respiratory Disease (CLRD) in 2020 and Their Health Care Expenditures in 2020 by Type of Services and Age Group eTable 2. Estimated Coefficients of the Econometric Models for the Impact of Cigarette Smoking on Health Care Utilization Among US Adults Aged 35+ Years With Chronic Lower Respiratory Disease: National Health Interview Survey, 2014-2018 eTable 3. Number of Smokers Among US Adults (Aged 35+ Years) With Chronic Lower Respiratory Disease (CLRD) in 2020 by Age Group eTable 4. Sensitivity Analysis Which Allows for the Negative SAF Values: Annual Cigarette Smoking-Attributable Health Care Expenditures (SAHEs) in 2020 by Type of Health Services, Smoking Status, and Age Group for US Adults Aged 35+ Years With Chronic Lower Respiratory Disease (CLRD) [file jamanetwopen-e2413869-s001.pdf]

Supplemental Online Content

Gu D, Sung H-Y, Calfee CS, Wang Y, Yao T, Max W. Smoking-attributable health care expenditures for US adults with chronic lower respiratory disease. *JAMA Netw Open*. 2024;7(5):e2413869. doi:10.1001/jamanetworkopen.2024.13869

eMethods. Supplemental Methods

eReferences

eTable 1. Number and Percentage of US Adults (Aged 35+ Years) Identified to Have Chronic Lower Respiratory Disease (CLRD) in 2020 and Their Health Care Expenditures in 2020 by Type of Services and Age Group

eTable 2. Estimated Coefficients of the Econometric Models for the Impact of Cigarette Smoking on Health Care Utilization Among US Adults Aged 35+ Years With Chronic Lower Respiratory Disease: National Health Interview Survey, 2014-2018

eTable 3. Number of Smokers Among US Adults (Aged 35+ Years) With Chronic Lower Respiratory Disease (CLRD) in 2020 by Age Group

eTable 4. Sensitivity Analysis Which Allows for the Negative SAF Values: Annual Cigarette Smoking-Attributable Health Care Expenditures (SAHEs) in 2020 by Type of Health Services, Smoking Status, and Age Group for US Adults Aged 35+ Years With Chronic Lower Respiratory Disease (CLRD)

This supplemental material has been provided by the authors to give readers additional information about their work.

© 2024 Gu D et al. *JAMA Network Open.*

eMethods. Supplemental Methods

Specification and Estimation Method for the Econometric Models for Cigarette Smoking-Attributable Healthcare Utilization Adapting the models that Max and colleagues have developed and refined over the past 3 decades,[1-5] we analyzed the association between cigarette smoking and healthcare utilization among persons with chronic lower respiratory disease (CLRD) using a 9-eqution econometric model. Each equation or group of equations is described below in terms of the dependent variable, independent variables, and functional form.

Equation 1: *Propensity of Having Poor Health Status*[a] = f_1 (smoking status[b], X)

Equations 2-5: *Propensity of Having Positive Utilization for each of four types of healthcare utilization*[c] = f_2 (smoking status[b], X, Y, predicted propensity of having poor health status from Equation 1)

Equations 6-9: *Natural Logarithm of Utilization Level among adults with Positive Utilization for each of four types of healthcare utilization*[c] = f_3 (smoking status[b], X, Y, predicted propensity of having poor health status from Equation 1)

where:

X = covariates (including gender, age, race/ethnicity, educational level, marital status, region of residence, body weight status, and binge drinking status);

Y = covariates (including X, and poverty status and health insurance coverage);

f_1 = ordered probit function;

f_2 = probit function;

© 2024 Gu D et al. *JAMA Network Open.*

f_3 = log-linear least squares;

[a]Self-reported health status is measured as an ordinal variable ranging from 0 (excellent), 1 (very good), 2 (good), 3 (fair), to 4 (poor);

[b]Smoking status is categorized into four groups: current smokers, former smokers who quit <15 years ago (i.e., 'shorter-term quitters'), former smokers who quit 15+ years ago (i.e., 'long-term quitters'), and never smokers (reference group);

[c]Four types of healthcare utilization include inpatient care measured by the number of hospital nights in the past 12 months, the number of ED visits in the past 12 months, the number of physician visits in the past 2 weeks, and the number of home health visits in the past 2 weeks.

Equation 1 was estimated as a an ordered probit model, Equations 2-5 were estimated as a probit model, and Equations 6-9 were equation were estimated by ordinary least squares. The statistical significance for the estimated coefficient was determined by chi-square test for the ordered Probit model in Equation 1 and the Probit models in Equations 2-5, and t-test for the log-linear model in Equations 5-9.

Based on the estimated 9-equation econometric model, we calculated two sets of predicted healthcare utilization: one for the factual case and one for a counterfactual case. In the factual case, the set of predicted utilization was calculated for smokers. In the counterfactual case, the set of predicted expenditures was calculated for "hypothetical never-smoking smokers", who are identical to smokers in every way except that they are assumed to be never smokers.[4,6,7] The difference between the factual and the counterfactual predictions is the excess utilization attributed to cigarette smoking. The ratio of the excess utilization to the sum of predicted values for all individuals (including smokers and never smokers) from the factual case is the smoking-attributable fraction

© 2024 Gu D et al. *JAMA Network Open.*

(SAF) of health utilization. The ratio of the mean predicted utilization for smokers (or ‘hypothetical never-smoking smokers”) to the mean predicted expenditures for never smokers is the relative risk (RR) of healthcare utilization for smokers (or ‘hypothetical never-smoking smokers”).

The SAF indicates the proportion of healthcare utilization that could be attributed to smoking. In this study, there are 3 categories of smokers: 1) current smokers, 2) former smokers who quit <15 years ago, and 3) former smokers who quit 15+ years ago. Following the previous studies,[4,5] the SAF of healthcare utilization for category *i* smokers can be expressed as a function of smoking prevalence and RRs as shown in the formula below.

$$SAF_i = \frac{P_i * (RR_i - RR_{i \to n})}{P_n + P_1 * (RR_1) + P_2 * (RR_2) + P_3 * (RR_3)} \qquad \textbf{(Equation 10)}$$

where:

P_n = prevalence of never smokers;

P_i = prevalence of category *i* smokers;

P_1, P_2, P_3= prevalence of current smokers, prevalence of former smokers who quit <15 years ago, prevalence of former smokers who quit 15+ years ago;

RR_i = relative risk of healthcare utilization type for category *i* smokers;

$RR_{i \to n}$ = relative risk of healthcare utilization for ‘hypothetical never-smoking smoker’ category *i* smokers.

© 2024 Gu D et al. *JAMA Network Open.*

eReferences

1. Miller LS, Zhang X, Novotny T, Rice DP, Max W. State estimates of Medicaid expenditures attributable to cigarette smoking, fiscal year 1993. Public health reports. 1998;113(2):140.
2. Max W, Rice D, Sung H, Zhang X, Miller L. The economic burden of smoking in California. Tobacco Control. 2004;13(3):264-267.
3. Max W, Sung HY, Tucker LY, Stark B. The disproportionate cost of smoking for African Americans in California. Am J Public Health. 2010;100(1):152-158.
4. Max W, Sung HY, Lightwood J. The impact of changes in tobacco control funding on healthcare expenditures in California, 2012-2016. Tob Control. 2013;22(e1):e10-15.
5. Max W, Sung HY, Shi Y, Stark B. The Cost of Smoking in California, 2009. San Francisco, CA: Institute for Health & Aging, University of California, San Francisco. 2014. https://trdrp.org/files/cost-smoking-ca-final-report.pdf (Accessed July 15, 2023)
6. Max W, Sung HY, Shi Y, Stark B. The Cost of Smoking in California. Nicotine Tob Res. 2016;18(5):1222-1229.
7. Wang Y, Sung HY, Lightwood J, Yao T, Max WB. Healthcare utilisation and expenditures attributable to current e-cigarette use among US adults. Tob Control. 2022.

© 2024 Gu D et al. *JAMA Network Open.*

eTable 1. Number and Percentage of US Adults (Aged 35+ Years) Identified to Have Chronic Lower Respiratory Disease (CLRD) in 2020 and Their Health Care Expenditures in 2020 by Type of Services and Age Group

Variable	Age 35-64			Age 65+			Adults with CLRD, both age groups
	All adults	Adults with CLRD		All adults	Adults with CLRD		
	Number or amount	Number or amount	Row %	Number or amount	Number or amount	Row %	Number or amount
**Number of persons**[a]	123,811,537	11,211,222	9.1%	58,265,600	7,561,909	13.0%	18,773,131
**Amount of annual healthcare expenditures**[a] **by type of services (in billion $):**							
Inpatient care	$190.99	$38.23	20.0%	$168.10	$30.92	18.4%	$69.15
ED visits	$29.45	$6.38	21.7%	$14.06	$2.34	16.6%	$8.72
Physician visits	$356.84	$60.09	16.9%	$243.91	$40.16	16.5%	$100.25
Home health visits	$29.43	$6.83	23.2%	$ 71.82	$17.24	24.0%	$24.09
**All four types**	$606.71	$111.52	18.4%	$497.89	$90.67	18.21%	**$202.19**

Notes: ED = Emergency department. All the numbers are estimated from the weighted analysis.

[a]Estimated using data from the 2020 Medical Expenditure Panel Survey.

© 2024 Gu D et al. *JAMA Network Open.*

eTable 2. Estimated Coefficients of the Econometric Models for the Impact of Cigarette Smoking on Health Care Utilization Among US Adults Aged 35+ Years With Chronic Lower Respiratory Disease: National Health Interview Survey, 2014-2018 (N=13,017)

Independent variables in the model by age group	Ordered Probit model[a] on health status	Two-part model[b] on hospital nights		Two-part model[b] on ED visits		Two-part model[b] on physician visits		Two-part model[b] on home health visits	
		1st part (Probit model)	2nd part (log-linear model)	1st part (Probit model)	2nd part (log-linear model)	1st part (Probit model)	2nd part (log-linear model)	1st part (Probit model)	2nd part (log-linear model)
**Age 35-64:**									
Sample size	7,400	7,400	1,395	7,400	2,991	7,400	2,816	7,400	259
Current smoker	0.542***	-0.065	-0.188*	0.078*	-0.053	-0.145	-0.087***	-0.292**	-0.257
<15-year quitters	0.327***	0.115*	0.122	0.063	-0.044	0.059	-0.103***	-0.011	-0.151
15+year quitters	0.099*	-0.001	-0.074	0.019	-0.014	-0.011	-0.025	-0.154	-0.372
Never smoker (Reference)									
Predicted health status	N/A	0.317***	0.273***	0.239***	0.127***	0.188***	0.103***	0.397***	0.014
**Age 65+:**									
Sample size	5,617	5,617	1,484	5,617	2,140	5,617	2,429	5,617	466
Current smoker	0.247***	-0.069	-0.054	-.0157**	-0.093*	-0.082	-0.044	0.175	0.603***
<15-year quitters	0.239***	0.205***	0.168*	0.069	-0.134***	-0.003	-0.021	0.269**	0.417**
15+year quitters	-0.076*	-0.011	0.019	0.033	-0.058	0.210***	0.004	0.022	-0.081
Never smoker (Reference)									
Predicted health status	N/A	0.311***	0.263***	0.297***	0.159***	0.217***	0.075***	0.392***	0.132**

Notes. ED=Emergency department. *** Significant at two-tailed p <.001; ** Significant at two-tailed p <.01; * Significant at two-tailed p <.05. The statistical significance for the estimated coefficient was determined by chi-square test for the ordered Probit models and Probit models, and t-test for the log-linear models.

[a]In addition to smoking status, the model also controlled for gender, age, race/ethnicity, education, marital status, region of residence, body weight status, and binge drinking status.

[b]In addition to smoking status and predicted health status, the model also controlled for gender, age, race/ethnicity, education, marital status, region of residence, body weight status, binge drinking status, poverty status, and health insurance coverage status. The first part of the two-part model was estimated as a probit regression, and the second part of the two-part model was estimated as a log-linear regression.

© 2024 Gu D et al. *JAMA Network Open.*

eTable 3. Number of Smokers Among US adults (Aged 35+ Years) With Chronic Lower Respiratory Disease (CLRD) in 2020 by Age Group

Cigarette smoking status	Number of smokers[a]	
	Age 35-64	Age 65+
**Current smokers**	**3,508,504**	**1,451,033**
**Former smokers**	**3,496,790**	**4,104,904**
< 15-year quitters	2,254,447	1,684,758
15+ year quitters	1,242,343	2,420,146
**Never smokers**	**4,205,928**	**2,005,972**

Notes: All the numbers are estimated from the weighted analysis.

[a]Calculated by multiplying the 2020 smoking prevalence rates (shown in Table 3) by the population size for adults with CLRD in 2020 (shown in eTable 1).

© 2024 Gu D et al. *JAMA Network Open.*

eTable 4. Sensitivity Analysis Which Allows for the Negative SAF Values[a]: Annual Cigarette Smoking-Attributable Healthcare Expenditures (SAHEs) in 2020 by Type of Health Services, Smoking Status, and Age Group for US Adults Aged 35+ Years With Chronic Lower Respiratory Disease (CLRD)

Subgroups	SAHE[c] by type of health services ($[b], million)				Total SAHE ($[b], billion)	Per-smoker SAHE[d] ($[b])
	Inpatient care	ED visits	Physician visits	Home health visits		
**Age 35-64 group:**						
**Current smokers**	**5,240**	**419**	**2,936**	**1,062**	**9.7**	**2,752**
**Former smokers**	**2,017**	**166**	**1,318**	**400**	**3.9**	**1,083**
<15-year quitters	1,765	146	1,131	346	3.4	1,502
15+ year quitters	252	20	187	54	0.5	412
**All smokers**	**7,257 (53.5%)**	**585 (4.3%)**	**4,254 (31.4%)**	**1,462 (10.8%)**	**13.6 (100.0%)**	**2,010**
**Age 65+ group:**						
**Current smokers**	**1,211**	**63**	**636**	**563**	**2.5**	**1,704**
**Former smokers**	**688**	**38**	**389**	**305**	**1.4**	**346**
<15-year quitters	1,290	69	715	726	2.8	1,662
15+ year quitters	-602	-31	-327	-420	-1.4	-570
**All smokers**	**1,899 (47.5%)**	**102 (2.6%)**	**1,024 (25.5%)**	**868 (24.4%)**	**3.9 (100.0%)**	**701**

Notes. ED = Emergency department; SAF = smoking-attributable fraction. The number in parentheses denotes percent distribution.

[a]Based on the negative values originally estimated from the econometric model: -1.9%, -1.3%, -0.8%, and -2.4% for inpatient care, ED visits, physician visits, and home health visits.

[b]In 2020 dollar.

[c]Derived by multiplying the SAF values (shown in Table 4) by annual healthcare expenditures among adults with CLRD (shown in eTable 1 in Supplement 1) for each type of health services and age group.

[d]Derived by dividing total SAHE by the number of smokers with CLRD for each smoking status category (shown in eTable 3 in Supplement 1).

© 2024 Gu D et al. *JAMA Network Open.*
