# Supplementary material for: Smoking-Attributable Health Care Expenditures for US Adults With Chronic Lower Respiratory Disease
Source: JAMA Netw Open. 2024 May 30;7(5):e2413869. doi: 10.1001/jamanetworkopen.2024.13869 (PMC11140527; doi:10.1001/jamanetworkopen.2024.13869)
Supplement: Supplement 2. — Data Sharing Statement [file jamanetwopen-e2413869-s002.pdf]

Data Sharing Statement

Gu. Smoking-Attributable Health Care Expenditures for US Adults With Chronic Lower Respiratory Disease. *JAMA Netw Open*. Published May 30, 2024. doi:10.1001/jamanetworkopen.2024.13869

Data
Data available: No

Additional Information
Explanation for why data not available: Data for this study are available from pub-use files of National Health Interview Surveys and Medical Expenditure Panel Survey.
